# Dysregulated EGFR pathway in serum in early-stage breast cancer patients: A case control study

**DOI:** 10.1038/s41598-020-63375-z

**Published:** 2020-04-21

**Authors:** Ina Mathilde Kjær, Dorte Aalund Olsen, Ivan Brandslund, Troels Bechmann, Erik Hugger Jakobsen, Søren Bie Bogh, Jonna Skov Madsen

**Affiliations:** 1 Department of Biochemistry and Immunology, Lillebaelt Hospital, University Hospital of Southern Denmark, Vejle, Denmark; 2 Department of Regional Health Research, Faculty of Health Sciences, University of Southern Denmark, Odense, Denmark; 3 Department of Oncology, Lillebaelt Hospital, University Hospital of Southern Denmark, Vejle, Denmark; 4 OPEN, Open Patient data Explorative Network, Department of Clinical Research, University of Southern Denmark, Odense, Denmark

**Keywords:** Biomarkers, Oncology

## Abstract

The epidermal growth factor receptor (EGFR) and its ligands are involved in cancer pathogenesis and they might serve as circulating biomarkers. The current study aims to investigate if abnormal pre-treatment serum levels of EGFR and EGFR ligands are present in women with early-stage breast cancer and if up- or downregulation of EGFR and EGFR ligands occur in defined patient subgroups. Pre-treatment serum samples were obtained from 311 women with newly diagnosed early-stage breast cancer and from 419 healthy women and analysed for EGFR and the ligands: Epidermal growth factor (EGF), heparin-binding epidermal growth factor (HBEGF), betacellulin (BTC), amphiregulin (AREG), and transforming growth factor α (TGF-α). Previously, age-dependent 95% reference intervals for EGFR and the EGFR ligands have been established based on the healthy women population. S-EGFR, S-EGF, S-HBEGF, S-AREG, and S-TGFα were all significantly different in women with breast cancer compared to healthy women (p < 0.05). Elevated S-EGFR, according to the reference intervals, was present in 11.3% of breast cancer patients, whereas decreased S-EGF was found in 11.6%. Elevated S-EGFR was associated with estrogen receptor positivity of tumor (ER+) and a subgroup of ER + breast cancer patients showed markedly elevated S-EGFR (>120 ng/mL).

## Introduction

In women, breast cancer is the most frequent cancer and the leading cause of cancer deaths worldwide^1^. Members of the epidermal growth factor receptor (EGFR) family and their ligands are involved in cancer pathogenesis^2^. Ligands activating EGFR include epidermal growth factor (EGF), heparin-binding epidermal growth factor (HBEGF), amphiregulin (AREG), transforming growth factor α (TGF-α), and betacellulin (BTC)^2^. EGFR ligands are synthesized as transmembrane precursors and released after extracellular domain cleavage^3^. The ligands activate EGFR with diverging affinity and specificity^4^. Breast cancer is known to be a heterogeneous disease in terms of histological subtypes and expression of the estrogen receptor (ER) and human epidermal growth factor receptor 2 (HER2)^5,6^. Furthermore, dysregulation of EGFR and EGFR ligands in breast cancer tumors has been reported^7^. Treatments targeting HER2 and ER have radically improved the overall prognosis for breast cancer patients^6,8^. Even so, heterogeneity constitutes a substantial challenge, as lack of response to treatment and acquired resistance to treatment are common among breast cancer patients^9^. The EGFR and ER signaling pathways are known to interact through a complex crosstalk and this crosstalk plays an important role in breast cancer pathogenesis^10^. There are indications that EGFR and EGFR ligands might be involved in resistance to both antiestrogen treatment and HER2-targeted treatment^11,12^. Disease related up- or downregulation of EGFR and EGFR ligands in blood in breast cancer has been investigated in several case-control studies. Most studies, however, included few study participants and report contradictory results^13–30^. Few of these studies reported data on newly diagnosed breast cancer patients^18,19,23–26^. None of the studies evaluated both EGFR and the EGFR ligands collectively. Also, BTC has not previously been investigated in blood in a group of breast cancer patients. The prognostic and predictive value of EGFR and EGFR ligands in blood in breast cancer is furthermore still unclear^31^. Highly sensitive methods for measuring EGFR ligands have recently been developed^32,33^ which has enabled detection of EGFR ligands with low abundance in the blood. We have previously established age-dependent reference intervals for EGFR and EGFR ligands^33^ which enables evaluation of disease related up- or downregulation of EGFR and EGFR ligands in breast cancer patients against reference intervals. The aim of the present study was to investigate serum levels of EGFR and EGFR ligands in early-stage breast cancer patients at the time of primary diagnosis prior to surgery and to evaluate if disease related up- or downregulation of EGFR and EGFR ligands occurs in specific subgroups of breast cancer patients.

## Material and method

### Study population and samples

A total of 383 breast cancer patients were enrolled in the study from 2004 until 2013. All patients were enrolled in the study shortly after diagnosis of primary breast cancer at Lillebaelt Hospital, University Hospital of Southern Denmark, Vejle, Denmark. Blood samples were obtained before primary breast cancer surgery. All samples were stored at −80 °C in a biobank at Lillebaelt Hospital. All patients gave written informed consent to participate in the study approved by The Regional Committees on Health Research Ethics for Southern Denmark (S-VF-20040101). Approval for analysis of EGFR and EGFR ligands was also obtained (S-20170119). The study was approved by the Danish Data Protection Agency (journal number 8/56003) and conducted in accordance to the Helsinki Declaration. Serum samples from a total of 419 healthy women aged 26–78 years were included in the healthy control group. The serum samples were retrieved from healthy female controls from Vejle Diabetes Biobank in the period 2007–2010 as described in previous studies by Petersen et al. and Kjær *et al*.^33,34^. Age dependent 95% reference intervals for EGFR and EGFR ligands in serum in women have been determined based on these samples in a previous study^33^.

### Assays

Levels of EGFR and the ligands EGF, HBEGF, AREG, TGF-α, and BTC were measured in all serum samples. The assays used to analyse the serum samples in the present study have been described in detail in previous studies by Kjær *et al*.^33^ and Olsen *et al*.^32^. S-EGFR and S-EGF were analysed using ELISA tests, whereas S-HBEGF, S-AREG, S-TGFα, and S-BTC were analysed on the Simoa platform; A highly sensitive immunoassay, enabling quantification of very low concentrations of the biomarkers^35^. Serum samples from breast cancer patients and healthy controls were mixed so every run included samples of both types. At least three assay controls were included in each run and demonstrated the following total coefficients of variation (CV%): S-EGFR 8–17%, S-EGF 8–12%, S-HBEGF 15–29%, S-AREG 12–21%, S-TGFα 8–14%, and S-BTC 11–25%.

ER-status of the breast cancer tumor was determined using immunohistochemical staining (IHC) detecting the ER-α isoform. An anti-human ER monoclonal antibody (clone 1D5; DakoCytomation) were used. Tumors were considered ER-positive if ≥10% of nuclei were stained according to contemporary guidelines. HER2-status of the tumor was determined using IHC (HercepTest (DakoCytomation, Glostrup, Denmark)) and considered HER2-positive in cases with IHC 3+ and HER2-negative in cases with IHC 0 or IHC 1+. IHC 2+ were considered equivocal and fluorescence *in situ* hybridization (FISH) were performed using HER2 FISH pharmDx kit (DakoCytomation, Glostrup, Denmark). Cases were considered HER2-positive when the ratio between HER2-gene copy number and the chromosome 17 centromer was ≥2.0.

### Statistical methods

First, age-adjusted linear regression analyses evaluating associations between biomarker level and status as either breast cancer patient or healthy control were carried out for each biomarker. Residual plots were evaluated. The distribution of the residuals of the S-EGF model approximated a Gaussian distribution. The residuals of the S-EGFR, S-HBEGF, S-AREG, S-TGFα, and S-BTC models approximated a Gaussian distribution after logarithmic transformation. Robust standard errors were applied to all models to account for heteroscedasticity in data. Second, the number and fractions of breast cancer patients with biomarker levels above, within, and below the reference intervals were evaluated using the upper and lower limits of age-dependent 95% reference intervals as cutoffs^33^. Third, we evaluated associations between elevated S-EGFR and decreased S-EGF, respectively, in the breast cancer patients and baseline clinicopathological characteristics using Pearson’s Chi square test. Probability value of <0.05 was considered significant. Finally, in order to evaluate the distribution of observations outside the reference intervals in healthy individuals and breast cancer patients, the number and fractions of individuals who had between zero and six biomarker results classified as abnormal according to the reference intervals, were evaluated. The statistical analyses were conducted using Stata IC 15 software package (StataCorp. 2017. Stata Statistical Software: Release 15. College Station, TX: StataCorp LLC).

## Results

**Figure 1:**
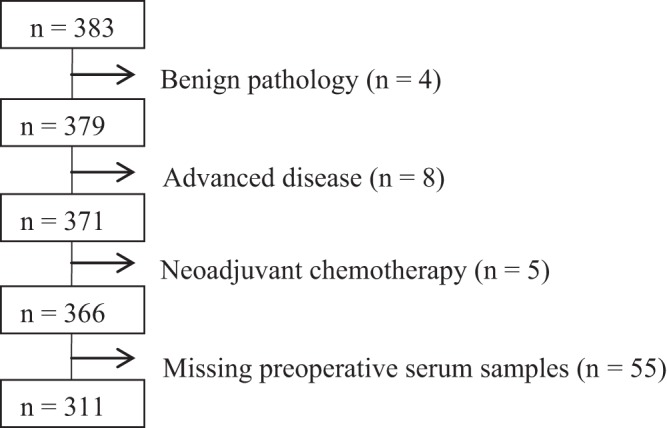
Diagram outlining the exclusion of patients.

**Figure 2:**
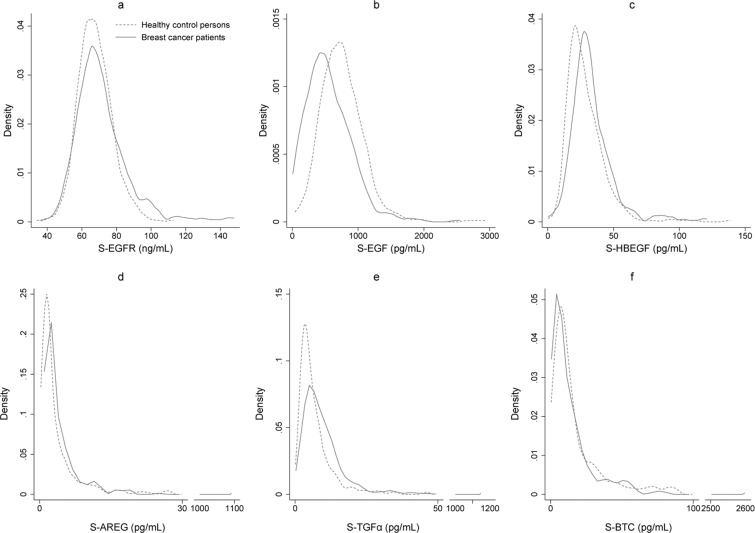
Kernel density plots of S-EGFR (**a**), S-EGF (**b**), S-HBEGF (**c**), S-AREG (**d**), S-TGFα (**e**), and S-BTC (**f**) levels in healthy female controls and early-stage breast cancer patients.

**Figure 3:**
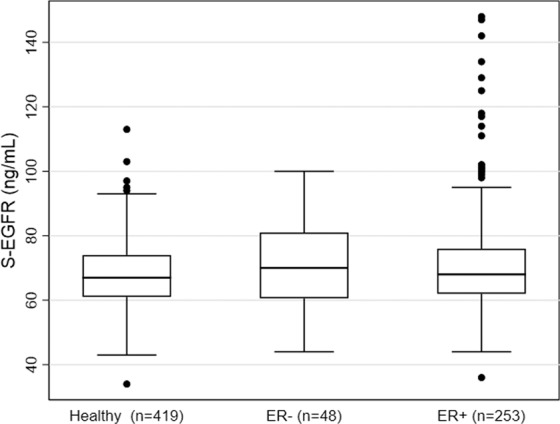
Levels of S-EGFR in healthy female controls and estrogen receptor positive (ER+) and estrogen receptor negative (ER−) early-stage breast cancer patients. Results are presented as box plots, where the ends of the boxes define the 25th and 75th percentiles, with a line at the median and error bars that define the upper and lower adjacent values.

Of the 383 breast cancer patients included in the study, a total of 17 patients were excluded due to benign breast disease (n = 4), advanced breast cancer (n = 8), or because they received neoadjuvant treatment (n = 5). Of the remaining 366 patients with early-stage breast cancer, 55 patients did not have available preoperative blood samples, resulting in a total of 311 breast cancer patients included in the present study (Fig. 1). The clinicopathological baseline characteristics of the breast cancer patients are presented in Table 1. The breast cancer patients were significantly younger than the healthy controls (p < 0.001), thus, age was included in the statistical analysis. The characteristics of the study population of breast cancer patients were compared to patients with early-stage breast cancer registered in the National Danish Breast Cancer Database (DBCG database) in the period 2005–2009^36^ (Appendix 1). Comparison of the groups using Pearson’s Chi square test showed that the study population in general was comparable to the national database. No significant differences were found regarding ER-status (p = 0.3) and nodal status (p = 0.4). Significant difference was found between the groups regarding HER2-status (p < 0.001) due to a higher fraction of patients with unknown HER2-status in the national database. When excluding patients with unknown HER2-status, there was no difference in HER2-status between the study population and the patients from the national database (p = 0.9). The breast cancer patients in the study population were significantly younger than the patients in the national database and there was a significantly lower incidence of grade I tumors in our population. Tumor size and histological type were shown to differ significantly; however, differences between the groups were quantitatively small. Based on this comparison, the current study population is considered representative for women with early-stage breast cancer. Median and interquartile range of the concentrations of each of the six biomarkers in healthy women and breast cancer patients are shown in Table 2. Age-adjusted linear regressions showed significantly lower levels of S-EGF in women with breast cancer as compared to healthy women (p < 0.001), whereas levels of S-EGFR (p < 0.001), S-HBEGF (p < 0.001), S-AREG (p = 0.002), and S-TGFα (p < 0.001) were significantly higher in women with breast cancer as compared to healthy women. No difference between the groups was found regarding S-BTC. Age had a significant effect on the models for all biomarkers, except S-HBEGF. Results are presented in Table 2. Figure 2 depicts the distributions of the concentrations of S-EGFR (2a), S-EGF (2b), S-HBEGF (2c), S-AREG (2d), S-TGFα (2e), and S-BTC (2f) in breast cancer patients and healthy controls, respectively. The number and fraction of breast cancer patients with levels above, within, and below the age-dependent 95% reference intervals^33^ for each of the six biomarkers are presented in Table 3. Elevated S-EGFR was observed in 11.3% of the breast cancer patients, whereas decreased S-EGF was observed in 11.6% of the patients. For the remaining ligands, less than 5.5% of the breast cancer patients had abnormal levels according to the reference intervals. In order to investigate if EGFR-pathway dysregulation occurs in specific subgroups of breast cancer patients, associations between elevated S-EGFR and decreased S-EGF and clinicopathological characteristics were evaluated. Results are presented in Table 4. Elevated S-EGFR was significantly associated to ER-positivity of the tumor. Figure 3 illustrates, that a subgroup of patients with ER-positive tumors had S-EGFR levels above 120 ng/mL. Neither patients with ER-negative tumors nor healthy controls had similarly high S-EGFR levels. No associations with dysregulation of the five EGFR ligands were observed among the ER-positive patients with elevated S-EGFR (data not reported). No association between decreased S-EGF and clinicopathological characteristics was observed, as shown in Table 4. In order to evaluate the distribution of observations outside the reference intervals in healthy individuals and breast cancer patients, we evaluated the number of individuals who had between zero and six biomarkers diverging from the reference intervals. A total of 79 (25%) breast cancer patients and 60 (14%) healthy controls had one abnormal biomarker, whereas two abnormal biomarker levels were observed among 23 (7.4%) breast cancer patients and 15 (3.6%) healthy controls. Three abnormal biomarker levels were observed among 6 (1.9%) breast cancer patients and 8 (1.9%) healthy controls. None of the healthy individuals had more than three of the biomarker levels outside the reference intervals. In comparison, five breast cancer patients (1.6%) had four or more biomarker levels outside the reference intervals. In general, the percentage of individuals with abnormal levels of one or more of EGFR or EGFR ligands in serum were found to be nearly twice as high in the breast cancer group compared to the healthy control group (35.9% and 19.5%, respectively).

**Table 1:** Baseline characteristics of the early-stage breast cancer patients included in the study.

	Breast cancer patients, n (%)
n	311
Age at time of primary surgery	
<50 years	65 (21%)
50–69 years	197 (63%)
≥70 years	49 (16%)
Tumor size	
T1 ≤ 20 mm	175 (56%)
T2 > 20 ≤ 50 mm	132 (42%)
T3 > 50 mm	4 (1.3%)
Histological type^a^	
Ductal	276 (89%)
Lobular	15 (4.8%)
Other	20 (6.4%)
Tumor grade	
Grade I	71 (23%)
Grade II	141 (45%)
Grade III	78 (25%)
Unknown	21 (6.8%)
Lymph node metastasis	
N0 = 0 nodes	155 (50%)
N1 = 1–3 nodes	111 (36%)
N2–3 ≥ 4 nodes	45 (14%)
Estrogen receptor status	
Negative (<10%)	58 (19%)
Positive (10–100%)	253 (81%)
HER2 IHC/FISH status^b^	
Negative	248 (80%)
Positive	49 (16%)
Unknown	14 (4.5%)
Progesterone receptor status	
Negative (<10%)	91 (29%)
Positive (10–100%)	183 (59%)
Unknown	37 (12%)
Triple negative	
Yes	33 (11%)
No	227 (73%)
Unknown	51 (16%)

^a^In cases where more than one histological type was detected in the tumor, the histological type was considered to be ductal if the tumor was either both ductal and lobular or ductal and other. ^b^HER2 IHC/FISH: Status of human epidermal growth factor receptor 2 (HER2) in breast cancer tumor evaluated by immunohistochemistry (IHC) and fluorescence *in situ* hybridization (FISH). Positive: IHC 3+ or IHC 2+ and FISH ≥ 2. Negative: IHC 0 or IHC 1+ or IHC 2+ and FISH < 2.

**Table 2:** Serum levels of EGFR and EGFR ligands in healthy female controls and early-stage breast cancer patients.

	Healthy female controls (n = 419), median (IQR)	Breast cancer patients (n = 311), median (IQR)	Based on logarithmic transformed data (with the exception of S-EGF)		
			Coefficient^a^	P-value^a^	95% CI^a^
S-EGFR, ng/mL	67 (61, 74)	68 (62, 78)	6.2%	<0.001	3.1%; 8.3%
S-EGF, pg/mL	723 (531, 925)	490 (314, 768)	−160	<0.001	−211; −110
S-HBEGF, pg/mL	25 (19, 35)	30 (24, 39)	17%	<0.001	8.3%; 27%
S-AREG, pg/mL	2.1 (1.2, 4.7)	2.6 (1.7, 5.2)	31%	0.002	11%; 54%
S-TGFα, pg/mL	5.1 (3.2, 8.8)	7.8 (4.8, 12)	57%	<0.001	36%; 80%
S-BTC, pg/mL	11 (5.7, 22)	8.3 (4.3, 19)	−18%	0.06	−34%; 1.0%

IQR: interquartile range, CI: confidence interval ^a^ Results of age-adjusted linear regressions evaluating if serum levels of EGFR and EGFR ligands are associated to breast cancer diagnosis or not. The coefficient and 95% CI are presented as absolute numbers for S-EGF, which was not logarithmic transformed in the regression model. All other biomarkers were used as logarithmic transformed variables, thus, the coefficient and 95% CI are presented as relative numbers. Robust standard errors were applied to all six regression models.

**Table 3:** Classifications of early-stage breast cancer patients according to age-dependent 95% reference intervals for EGFR and EGFR ligands.

	Classification		
	Below reference interval, n (%)	Within reference interval, n (%)	Above reference interval, n (%)
S-EGFR	8 (2.6%)	268 (86.2%)	35 (11.3%)
S-EGF	36 (11.6%)	264 (84.9%)	11 (3.5%)
S-HBEGF	6 (1.9%)	288 (93.2%)	15 (4.9%)
S-AREG	3 (1.0%)	301 (96.8%)	7 (2.3%)
S-TGFα	3 (1.0%)	297 (95.5%)	11 (3.5%)
S-BTC	17 (5.5%)	282 (90.7%)	12 (3.9%)

Numbers and percentages of breast cancer patients classified as having levels above, within and below the reference intervals for each biomarker. The upper and lower limits of age-dependent 95% reference intervals for EGFR and EGFR ligands published by Kjær *et al*. were used as cutoffs^33^.

**Table 4:** Associations between elevated S-EGFR and decreased S-EGF, respectively, in early-stage breast cancer patients and baseline clinicopathological characteristics.

	S-EGFR			S-EGF		
	Within or below reference interval n (%)	Above reference interval n (%)	P-value^c^	Below reference interval n (%)	Within or above reference interval n (%)	P-value^c^
n	276	35		36	275	
Age			0.53			0.015
<50 years	60 (22%)	5 (14%)		14 (39%)	51 (18%)	
50-69 years	172 (62%)	25 (71%)		19 (53%)	178 (65%)	
≥70 years	44 (16%)	5 (14%)		3 (8.3%)	46 (17%)	
Tumor size			0.03			0.66
T1 ≤20 mm	162 (59%)	13 (37%)		19 (53%)	156 (57%)	
T2 >20 ≤50 mm	110 (40%)	22 (63%)		17 (47%)	115 (42%)	
T3 >50 mm	4 (1.4%)	0 (0.0%)		0 (0.0%)	4 (1.5%)	
Histological type^a^			0.54			0.63
Ductal	246 (89%)	30 (86%)		33 (92%)	243 (88%)	
Lobular	12 (4.3%)	3 (8.6%)		2 (5.6%)	13 (4.7%)	
Other	18 (6.5%)	2 (5.7%)		1 (2.8%)	19 (6.9%)	
Tumor grade			0.74			0.43
Grade I	65 (24%)	6 (17%)		6 (17%)	65 (24%)	
Grade II	125 (45%)	16 (46%)		17 (47%)	124 (45%)	
Grade III	67 (24%)	11 (31%)		12 (33%)	66 (24%)	
Unknown	19 (6.9%)	2 (5.7%)		1 (2.8%)	20 (7.3%)	
Lymph node metastasis			0.47			0.51
N0 = 0 nodes	141 (51%)	14 (40%)		15 (42%)	140 (51%)	
N1 = 1-3 nodes	96 (35%)	15 (43%)		14 (39%)	97 (35%)	
N2-3 ≥4 nodes	39 (14%)	6 (17%)		7 (19%)	38 (14%)	
Estrogen receptor status			0.04			0.90
Negative (<10%)	56 (20%)	2 (5.7%)		7 (19%)	51 (18%)	
Positive (10-100%)	220 (80%)	33 (94%)		29 (81%)	224 (81%)	
HER2 IHC/FISH status^b^			0.37			0.93
Negative	218 (79%)	30 (86%)		28 (78%)	220 (80%)	
Positive	44 (16%)	5 (14%)		6 (17%)	43(16%)	
Unknown	14 (5.1%)	0 (0.0%)		2 (5.6%)	12 (4.4%)	
Progesterone receptor status			0.44			0.96
Negative (<10%)	84 (30%)	7 (20%)		10 (28%)	81 (29%)	
Positive (10-100%)	160 (58%)	23 (66%)		22 (61%)	161 (58%)	
Unknown	32 (12%)	5 (14%)		4 (11%)	33 (12%)	

^a^In cases where more than one histological type was detected in the tumor, the histological type was considered to be ductal if the tumor was either both ductal and lobular or ductal and other. ^b^HER2 IHC/FISH: Status of human epidermal growth factor receptor 2 (HER2) in breast cancer tumor evaluated by immunohistochemistry (IHC) and fluorescence *in situ* hybridization (FISH). Positive: IHC 3+ or IHC 2+ and FISH ≥ 2. Negative: IHC 0 or IHC 1+ or IHC 2+ and FISH < 2. ^c^The distributions are compared using Pearson’s chi squared test.

## Discussion

In the present study we demonstrated significant differences in levels of S-EGFR, S-EGF, S-HBEGF, S-AREG, and S-TGFα between women with early-stage breast cancer and healthy women. Furthermore, we identified subgroups of breast cancer patients with abnormal levels of S-EGFR and S-EGF according to previously established reference intervals. To our best knowledge, this study is the first to investigate blood levels of both EGFR and EGFR ligands in a large population of early-stage breast cancer patients compared to healthy controls. S-EGFR was markedly higher in the group of breast cancer patients than in healthy women, and, when evaluating against age-dependent 95% reference intervals^33^, we found that 11.3% of the breast cancer patients had elevated S-EGFR values. Elevated S-EGFR in the breast cancer patients was associated with ER-positivity of the tumor, and a subgroup of patients with ER-positive tumors and very high levels of S-EGFR (>120 ng/mL) were identified. Conversely, neither patients with ER-negative tumors nor healthy women had similarly high levels. Values of S-EGF were lower in breast cancer patients compared to the healthy control group, and 11.6% of the breast cancer patients had decreased S-EGF according to the age-dependent 95% reference intervals^33^. In contrast to what was found for S-EGFR, no association between S-EGF and clinicopathological characteristics was, however, observed. S-HBEGF, S-AREG, and S-TGFα-levels were higher in the group of women with breast cancer, compared to the healthy group, but only a limited number of patients showed increased serum levels for these parameters according to the reference intervals^33^. Regarding S-BTC, no difference between breast cancer patients and healthy women was observed. In general, it was nearly twice as frequent among breast cancer patients compared to healthy women to have abnormal levels of one or more of the EGFR or EGFR ligands. Furthermore, individuals with more than three of the biomarker levels outside the reference intervals were only observed among breast cancer patients. Overall, the results indicate EGFR-pathway dysregulation in subgroups of early-stage breast cancer patients that can be identified by measuring levels of EGFR and EGFR ligands in the blood. The causality behind these findings is unknown. One explanation might be tumor-related alteration of the expression of EGFR and EGFR ligands, which is reflected in the blood. Another explanation could be a general EGFR-pathway dysregulation driven by an endogenous or exogenous hormonal disturbance. A feedback mechanism might be causing the decreased S-EGF seen in some breast cancer cases. Furthermore, in the subgroup of breast cancer patients with elevated S-EGFR, a significant association to ER-positivity of the tumor was found, and some of the patients in this group had very high S-EGFR levels (≥120 ng/mL) (n = 6). This group might be of special interest as a crosstalk between EGFR and ER pathways is known^12^. It can be hypothesized that this subgroup of ER-positive patients might potentially benefit from EGFR-targeted treatment. The present study has several strengths. The study is based on a large and well described population of breast cancer patients and healthy controls and evaluates serum levels of both EGFR and EGFR ligands, enabling an extensive overview of blood levels of EGFR and EGFR ligands across the EGFR pathway in both healthy female controls and breast cancer patients. The EGFR and EGFR ligand reference intervals established in a previous study^33^ enables evaluation of levels of EGFR and EGFR ligands in breast cancer patients against reference intervals and not only by groups comparison, as done in previous studies. Furthermore, the population of breast cancer patients can be considered representative for early-stage breast cancer patients in Denmark. Finally, highly sensitive immunoassays were used to analyse the serum samples, enabling quantification of the biomarkers in nearly all serum samples included in the study. The analyses of patient samples in the present study were performed using the same assays used for the establishment of the applied reference intervals. The limitations of the present study include varying CV’s of the assays between 8–29%. If the biomarkers are to be used for individual patients in a clinical context, there is a need for improving the analytical performance of the assays. In the present study, only patients primarily diagnosed with non-advanced breast cancer were included. Thus, it is possible that the observed differences between EGFR and EGFR ligand levels in serum from breast cancer patients and healthy women would be even more pronounced if patients with all stages of disease at time of diagnosis were included. Also, the healthy female controls included in the study were considered healthy at the time of blood sampling, but it is unknown if any of them later developed or were diagnosed with breast cancer. If this was the case, excluding these individuals would, however, probably not reduce the observed differences between breast cancer patients and healthy controls reported in the present study. If anything, it would probably enhance the reported findings. The healthy female controls in the study were significantly younger than the breast cancer patients. This might potentially bias the results. Regressions were, however, adjusted for age and the reference intervals used as cut-offs were age-dependent reference intervals^33^. To provide an entire overview of EGFR ligand levels in serum in breast cancer patients, the two low-affinity ligands epigen and epiregulin^4,37^ should have been included in the study. Due to lack of antibodies of the desired quality we, however, refrained from investigating epigen and epiregulin in this study. In conclusion, the current study demonstrated differences in pre-treatment levels of S-EGFR, S-EGF, S-HBEGF, S-AREG, and S-TGFα s in early-stage breast cancer patients as compared to a healthy control group. Elevated S-EGFR, according to reference intervals, was found in 11.3% of the breast cancer patients and decreased S-EGF was found in 11.6% of breast cancer patients. A subgroup of breast cancer patients with ER-positive tumors and markedly elevated S-EGFR levels might be of particular interest as potential candidates for EGFR-targeted treatment. The findings of the present study is a stepping stone for further investigations of the clinical potential of using blood levels of EGFR and EGFR ligands in tailored treatment of breast cancer in order to improve outcome for the patients.

## Data Availability

The dataset contains sensitive data which were used under license for the current study, and is therefore not publicly available. Data are, however, available from the authors upon reasonable request and with permission from the relevant legal authorities under existing laws.

## Appendix 1 –

Comparison of study population of early-stage breast cancer patients to the cohort of early-stage breast cancer patients from the Danish Breast Cancer Group (DBCG) database (2005–2009)^36^

**Table:**

	Study population	DBCG cohort	p-value
n	311	18956	
Age at time of primary surgery			0.018
<50 years	65 (20.9%)	3323 (17.5%)	
50–69 years	197 (63.3%)	11437 (60.3%)	
≥70 years	49 (15.8%)	4196 (22.1%)	
Tumor size			0.02
T1 ≤ 20 mm	175 (56.3%)	11250 (59.4%)	
T2 > 20 ≤ 50 mm	132 (42.4%)	6918 (36.5%)	
T3 > 50 mm	4 (1.3%)	638 (3.4%)	
Unknown	—	150 (0.79%)	
Histological type			0.007
Ductal	276 (88.8%)	15582 (82.2%)	
Lobular	15 (4.8%)	1879 (9.9%)	
Other	20 (6.4%)	1363 (7.2%)	
Unknown	—	132 (0.7%)	
Tumor grade			<0.001
Grade I	71 (22.8%)	5535 (31.7%)	
Grade II	141 (45.3%)	7597 (43.5%)	
Grade III	78 (25.1%)	3885 (22.3%)	
Unknown	21 (6.8%)	444 (2.54%)	
Lymph node metastasis			0.4
N0 = 0 nodes	155 (49.8%)	9850 (52.0%)	
N1 = 1–3 nodes	111 (35.7%)	6109 (32.2%)	
N2-3 ≥ 4 nodes	45 (14.5%)	2997 (15.8%)	
Estrogen receptor status			0.3
Negative (<10%)	58 (18.6%)	3221 (17.0%)	
Positive (10–100%)	253 (81.4%)	15614 (82.4%)	
Unknown	—	121 (0.6%)	
HER2 IHC/FISH status			<0.001*
Negative	248 (79.8%)	11913 (62.9%)	
Positive	49 (15.8%)	2288 (12.1%)	
Unknown	14 (4.5%)	4755 (25.1%)	
Progesterone receptor status			
Negative (<10%)	91 (29.3%)	NR	
Positive (10–100%)	183 (58.8%)	NR	
Unknown	37 (11.9%)	NR	

NR: Not reported. *If the category “unknown” is left out of the Pearson chi-square, the p-value is 0.9.

## References

[1] Bray F (2018). Global cancer statistics 2018: GLOBOCAN estimates of incidence and mortality worldwide for 36 cancers in 185 countries. CA Cancer J Clin.

[2] Yarden Y, Sliwkowski MX (2001). Untangling the ErbB signalling network. Nat Rev Mol Cell Biol.

[3] Harris RC, Chung E, Coffey RJ (2003). EGF receptor ligands. Exp Cell Res.

[4] Jones JT, Akita RW, Sliwkowski MX (1999). Binding specificities and affinities of egf domains for ErbB receptors. FEBS Lett.

[5] Slamon DJ (1987). Human breast cancer: correlation of relapse and survival with amplification of the HER-2/neu oncogene. Science.

[6] Early Breast Cancer Trialists’ Collaborative, G. (2005). Effects of chemotherapy and hormonal therapy for early breast cancer on recurrence and 15-year survival: an overview of the randomised trials. Lancet.

[7] Olsen DA (2012). Increased concentrations of growth factors and activation of the EGFR system in breast cancer. Clin Chem Lab Med.

[8] Swain SM (2013). Pertuzumab, trastuzumab, and docetaxel for HER2-positive metastatic breast cancer (CLEOPATRA study): overall survival results from a randomised, double-blind, placebo-controlled, phase 3 study. Lancet Oncol.

[9] Tang Y, Wang Y, Kiani MF, Wang B (2016). Classification, Treatment Strategy, and Associated Drug Resistance in Breast Cancer. Clin Breast Cancer.

[10] Schiff R (2004). Cross-talk between estrogen receptor and growth factor pathways as a molecular target for overcoming endocrine resistance. Clin Cancer Res.

[11] Osborne CK, Schiff R (2011). Mechanisms of endocrine resistance in breast cancer. Annual review of medicine.

[12] Hynes NE, Lane HA (2005). ERBB receptors and cancer: the complexity of targeted inhibitors. Nat Rev Cancer.

[13] Lafky JM (2005). Serum soluble epidermal growth factor receptor concentrations decrease in postmenopausal metastatic breast cancer patients treated with letrozole. Cancer Res.

[14] Muller V (2006). Prognostic and predictive impact of soluble epidermal growth factor receptor (sEGFR) protein in the serum of patients treated with chemotherapy for metastatic breast cancer. Anticancer Res.

[15] Souder C (2006). Serum epidermal growth factor receptor/HER-2 predicts poor survival in patients with metastatic breast cancer. Cancer.

[16] Tas F, Bilgin E, Karabulut S, Duranyildiz D (2015). Clinical significance of serum epidermal growth factor receptor (EGFR) levels in patients with breast cancer. Cytokine.

[17] Banys-Paluchowski M (2017). Evaluation of serum epidermal growth factor receptor (EGFR) in correlation to circulating tumor cells in patients with metastatic breast cancer. Sci Rep.

[18] Bayo J, Castano MA, Rivera F, Navarro F (2018). Analysis of blood markers for early breast cancer diagnosis. Clin Transl Oncol.

[19] Asgeirsson KS (2007). Serum epidermal growth factor receptor and HER2 expression in primary and metastatic breast cancer patients. Breast Cancer Res.

[20] Kumar RR, Meenakshi A, Sivakumar N (2001). Enzyme immunoassay of human epidermal growth factor receptor (hEGFR). Hum Antibodies.

[21] Sandri MT (2007). Serum EGFR and serum HER-2/neu are useful predictive and prognostic markers in metastatic breast cancer patients treated with metronomic chemotherapy. Cancer.

[22] Pitteri SJ (2010). Detection of elevated plasma levels of epidermal growth factor receptor before breast cancer diagnosis among hormone therapy users. Cancer Res.

[23] Karlikova M (2016). Circulating Growth and Angiogenic Factors and Lymph Node Status in Early-stage Breast Cancer - A Pilot Study. Anticancer Res.

[24] Kim BK (2009). The multiplex bead array approach to identifying serum biomarkers associated with breast cancer. Breast Cancer Res.

[25] Kucera R (2011). Growth factors and breast tumors, comparison of selected growth factors with traditional tumor markers. Anticancer Res.

[26] Gonzalez RM, Daly DS, Tan R, Marks JR, Zangar RC (2011). Plasma biomarker profiles differ depending on breast cancer subtype but RANTES is consistently increased. Cancer Epidemiol Biomarkers Prev.

[27] Chakrabarty S, Huang S, Moskal TL, Fritsche HA (1994). Elevated serum levels of transforming growth factor-alpha in breast cancer patients. Cancer letters.

[28] Shetty, P. *et al*. Annexin A2 and its downstream IL-6 and HB-EGF as secretory biomarkers in the differential diagnosis of Her-2 negative breast cancer. *Ann Clin Biochem*, 10.1177/0004563216665867 (2016). 10.1177/0004563216665867 27496793

[29] Vlaicu P (2013). Monocytes/macrophages support mammary tumor invasivity by co-secreting lineage-specific EGFR ligands and a STAT3 activator. BMC Cancer.

[30] Perez EA (2002). A randomized phase II study of sequential docetaxel and doxorubicin/cyclophosphamide in patients with metastatic breast cancer. Annals of oncology: official journal of the European Society for Medical Oncology / ESMO.

[31] Kjaer IM, Bechmann T, Brandslund I, Madsen JS (2018). Prognostic and predictive value of EGFR and EGFR-ligands in blood of breast cancer patients: a systematic review. Clin Chem Lab Med.

[32] Olsen DA, Kjaer IM, Brandslund I (2018). Development of a three-plex single molecule immunoassay enabling measurement of the EGFR ligands amphiregulin, betacellulin and transforming growth factor alpha simultaneously in human serum samples. J Immunol Methods.

[33] Kjaer, I. M. *et al*. EGFR and EGFR ligands in serum in healthy women; reference intervals and age dependency. *Clin Chem Lab Med*, 10.1515/cclm-2019-0376 (2019). 10.1515/cclm-2019-0376 31323001

[34] Petersen ER (2016). Vejle Diabetes Biobank - a resource for studies of the etiologies of diabetes and its comorbidities. Clin Epidemiol.

[35] Rissin DM (2010). Single-molecule enzyme-linked immunosorbent assay detects serum proteins at subfemtomolar concentrations. Nat Biotechnol.

[36] Jensen MB, Ejlertsen B, Mouridsen HT, Christiansen P (2016). & Danish Breast Cancer Cooperative, G. Improvements in breast cancer survival between 1995 and 2012 in Denmark: The importance of earlier diagnosis and adjuvant treatment. Acta Oncol.

[37] Schneider MR, Yarden Y (2014). Structure and function of epigen, the last EGFR ligand. Semin Cell Dev Biol.

